# Urban-rural differences in the associated factors of severe under-5 child undernutrition based on the composite index of severe anthropometric failure (CISAF) in Bangladesh

**DOI:** 10.1186/s12889-021-12038-3

**Published:** 2021-11-23

**Authors:** Asibul Islam Anik, Mohammad Rocky Khan Chowdhury, Hafiz T. A. Khan, Md Nazrul Islam Mondal, Nirmala K. P. Perera, Manzur Kader

**Affiliations:** 1 Department of Public Health and Informatics, Bangabandhu Sheikh Mujib Medical University (BSMMU), Shahbag, Dhaka Bangladesh; 2 Department of Research and Evaluation, SAJIDA Foundation, Gulshan-1, Dhaka Bangladesh; 3 Department of Public Health, First Capital University of Bangladesh, Chuadanga, Bangladesh; 4 College of Nursing, Midwifery and Healthcare, University of West London, London, UK; 5 Department of Population Science and Human Resource Development, University of Rajshahi, Rajshahi, Bangladesh; 6 Nuffield Department of Orthopaedics, Rheumatology, and Musculoskeletal Sciences, University of Oxford, Oxford, UK; 7 Unit of Physiotherapy, Department of Health, Medicine and Caring Sciences (HMV), Linköping University, Linköping, Sweden; 8 Unit of Occupational Medicine, Institute of Environmental Medicine | Karolinska Institutet, Solnavägen 4, Torsplan floor 10, 113 65 Stockholm, Sweden

**Keywords:** CIAF, CISAF, Severe child undernutrition, Maternal & child health, Urban-rural disparity, Bangladesh

## Abstract

**Introduction:**

Severe undernutrition among under-5 children is usually assessed using single or conventional indicators (i.e., severe stunting, severe wasting, and/or severe underweight). But these conventional indicators partly overlap, thus not providing a comprehensive estimate of the proportion of malnourished children in the population. Incorporating all these conventional nutritional indicators, the Composite Index of Severe Anthropometric Failure (CSIAF) provides six different undernutrition measurements and estimates the overall burden of severe undernutrition with a more comprehensive view. This study applied the CISAF indicators to investigate the prevalence of severe under-5 child undernutrition in Bangladesh and its associated socioeconomic factors in the rural-urban context.

**Methods:**

This study extracted the children dataset from the 2017–18 Bangladesh Demographic Health Survey (BDHS), and the data of 7661 children aged under-5 were used for further analyses. CISAF was used to define severe undernutrition by aggregating conventional nutritional indicators. Bivariate analysis was applied to examine the proportional differences of variables between non-severe undernutrition and severe undernutrition group. The potential associated socioeconomic factors for severe undernutrition were identified using the adjusted model of logistic regression analysis.

**Results:**

The overall prevalence of severe undernutrition measured by CISAF among the children under-5 was 11.0% in Bangladesh (rural 11.5% vs urban 9.6%). The significant associated socioeconomic factors of severe undernutrition in rural areas were children born with small birth weight (AOR: 2.84), children from poorest households (AOR: 2.44), and children aged < 36 months, and children of uneducated mothers (AOR: 2.15). Similarly, in urban areas, factors like- children with small birth weight (AOR: 3.99), children of uneducated parents (AOR: 2.34), poorest households (APR: 2.40), underweight mothers (AOR: 1.58), mothers without postnatal care (AOR: 2.13), and children’s birth order ≥4 (AOR: 1.75), showed positive and significant association with severe under-5 undernutrition.

**Conclusion:**

Severe undernutrition among the under-5 children dominates in Bangladesh, especially in rural areas and the poorest urban families. More research should be conducted using such composite indices (like- CISAF) to depict the comprehensive scenario of severe undernutrition among the under-5 children and to address multi-sectoral intervening programs for eradicating severe child undernutrition.

**Supplementary Information:**

The online version contains supplementary material available at 10.1186/s12889-021-12038-3.

## Introduction

Undernutrition is one of the common causes of morbidity and mortality in children. Around 195 million children under-5 suffer from any form of undernutrition across the world [1, 2]. About half of all fatalities in children under-5 are attributable to undernutrition [3]. Asia is the home to over 70% of the world’s malnourished children, with India, Pakistan, and Bangladesh having the highest prevalence in this region [4]. In 2014, over 40% of Bangladeshi children under-5 experience undernutrition, with 18% experiencing severe undernutrition [5]. Children with severe undernutrition experience short- and long-term adverse physiological outcomes, such as poor physical growth, morbidity, inadequate cognitive development, and physical incapacity [6].

Undernutrition affects children for the rest of their lives, and its consequences are passed down from generation to generation. Since undernutrition is more prevalent among rural female children, these undernourished female children become stunted mothers in future. Then by giving birth to a stunted child, the circle continues to revolve [7]. Among the other South Asian countries, though Bangladesh made a significant achievement in declining the prevalence rate of stunting children from 1997 (60%) to 2018 (31%) [8], it is still inadequate to attain the target of ending all forms of undernutrition by 2030 (Sustainable Development Goal- 2, Target 2.2) [9]. There are considerable urban-rural disparities in terms of stunting in Bangladesh, with a higher prevalence among rural (33%) than urban (25%) children [8].

Along with the urban-rural disparities, other associated factors for undernutrition are shaped by people’s complex interactions with their social, cultural, economic, and environmental contexts. Social determinants of health provide a framework for understanding the inequalities in health risks and outcomes within and between populations [10]. Improper postnatal care (i.e., inadequate infant and young child feeding practices and inadequate access to health care); children’s low birth weight, frequent infections; parent’s least wealthy socioeconomic status (i.e., lower education level, unemployment, and poor wealth-index); and other household characteristics (i.e., unsafe drinking water, and lack of access to sanitation), etc. were found to be strongly associated with under-5 child undernutrition in different studies [5, 11–13]. However, current evidence on severe under-5 child undernutrition focused on single or may not provide a *convincing* (i.e., severe stunting, severe wasting, and/or severe underweight) and economic dimensions at single time points. But these conventional indicators partly overlap, thus not providing a comprehensive estimate of the proportion of malnourished children in the population [14].

By incorporating all these conventional nutritional indicators, the Composite Index of Anthropometric Failure (CIAF) provides six different undernutrition measurements and estimates the overall burden of undernutrition. Moreover, the CIAF accurately depicts a single aggregate proportion of all malnourished children in a population [14]. On the other hand, Vollmer et al. (2017) introduced an updated version of the CIAF, i.e., Composite Index of Severe Anthropometric Failure (CISAF), which provides a more comprehensive view of the extent and pattern of associated factors of severe undernutrition in resource-poor settings [15]. As per the CIAF, undernutrition was more prevalent among rural children (54%) than urban children (45%) in Bangladesh. Irrespective of urban-rural differences, sociodemographic characteristics of children, such as older age, mothers’ illiteracy, underweight mothers, and poorest wealth-index showed positive association with undernutrition [16, 17]. But rarely any study has applied the CISAF to explore the prevalence and the intricate interaction of individual, community, public policy, and environmental characteristics associated with severe under-5 child undernutrition in Bangladesh. Addressing this knowledge gap, this study used the CISAF to investigate the prevalence and associated socioeconomic factors of severe under-5 child undernutrition in Bangladesh’s rural and urban areas.

## Methodology

**Fig. 1:**
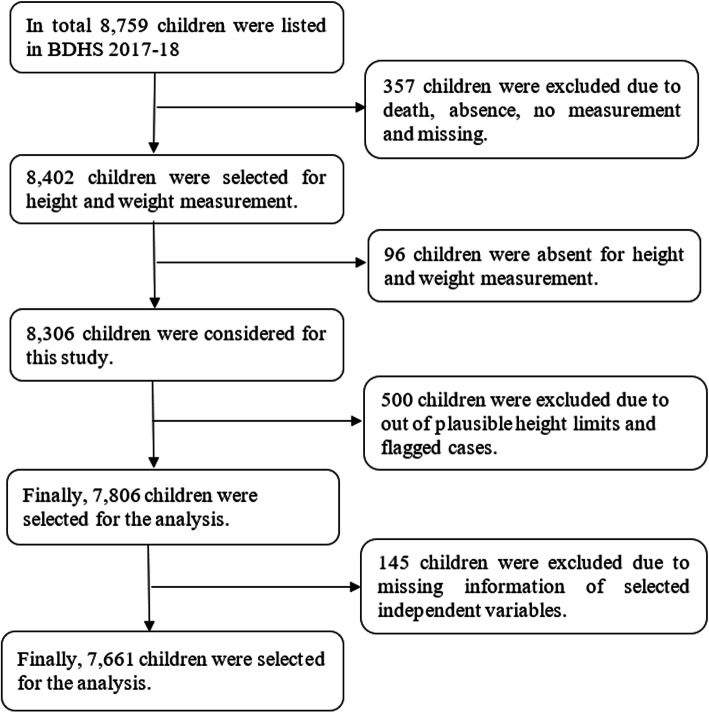
Sample size selection

We analysed the children dataset (BDKR7RDT) from 2017 to 18 of the Bangladesh Demographic Health Survey (BDHS). The average response rate was 99%. The BDHS uses two-stage stratified sampling techniques to select primary sampling units (PSUs) and households. During the first stage, PSUs or enumeration areas (EA) were designed based on the census survey 2011 conducted by the Bangladesh Bureau of Statistics. The probability proportional to the EA size technique was used to select PSUs. During the second stage, an equal probability systematic sampling technique was used to determine households from PSUs. Reproductive-aged women (15–49 years) were interviewed from each household. The BDHSs collect data on social and demographic factors, health, and nutritional factors from adults (male and female) residing in non-institutional dwellings. A standard questionnaire was used for data collection. For details of the survey questionnaire, sample design, data collection procedure (see BDHS reports 2017–18) [8]. The BDHS 2017–18 collected anthropometric data from 8759 under-5 children, and data from 7661 children were analysed (Fig. 1).

### Outcome variables

**Fig. 2:**
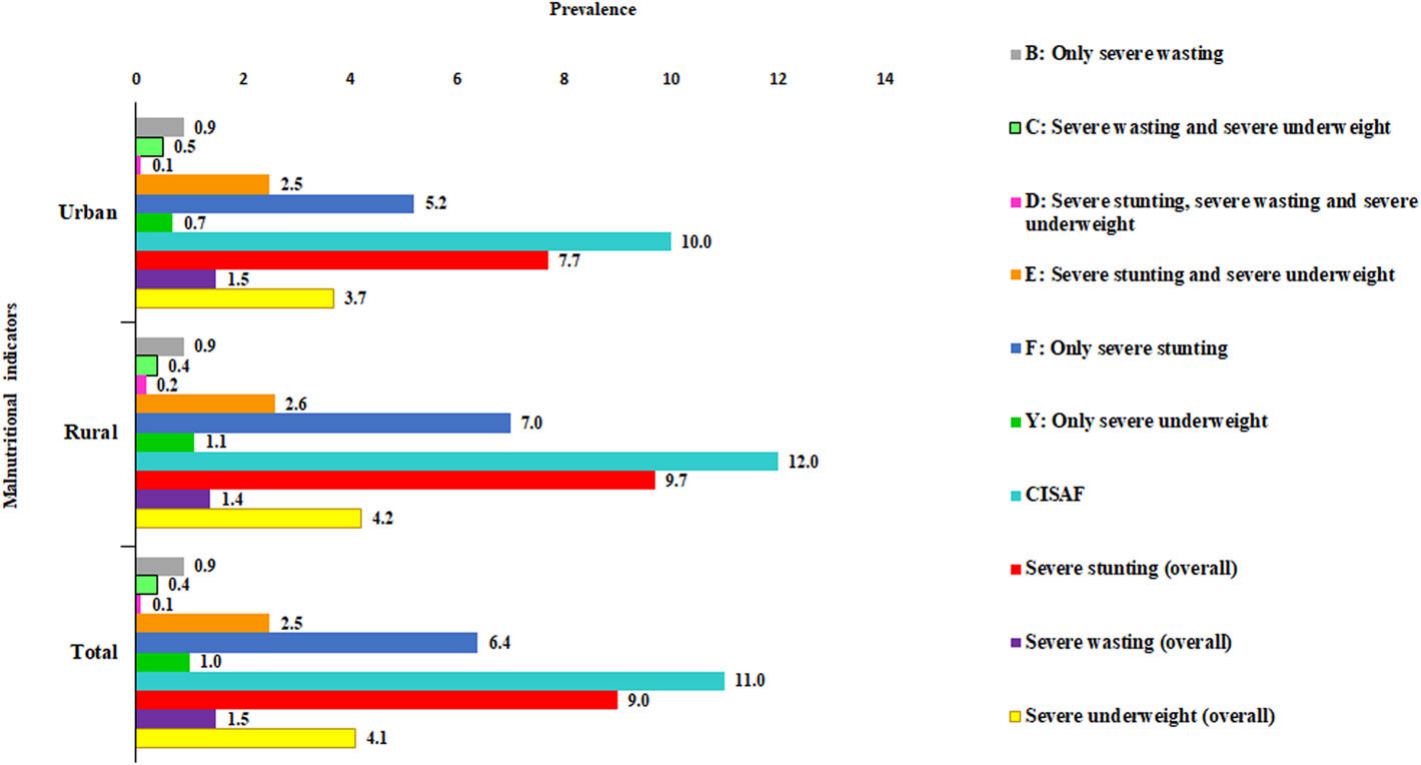
Prevalence of malnutritional indicators

Severe nutritional indicators for under-5 children were categorised into seven groups: (A) no severe failure; (B) severe wasting only; (C) severe wasting and severe underweight; (D) severe wasting and severe stunting and severe underweight; (E) severe stunting and severe underweight; (F) severe stunting only; and (G) severe underweight only (Table 1). A child is considered having severe undernutrition if she/he has any anthropometric failure from B to G (Fig. 2).

**Table 1:** Classification of children with severe anthropometric failure

Group name	Description	Severe Wasting	Severe Stunting	Severe Underweight
A	No severe failure	No	No	No
B	Severe wasting only	Yes	No	No
C	Severe wasting and severe underweight	Yes	No	Yes
D	Severe wasting, severe stunting and severe underweight	Yes	Yes	Yes
E	Severe stunting and severe underweight	No	Yes	Yes
F	Severe stunting only	No	Yes	No
G	Severe underweight only	No	No	Yes

The primary outcome measure was severe under-5 child undernutrition using the CISAF. A child was considered to be severely stunted and severely wasted and severely underweight if the z-scores of length/height-for-age, weight-for-height/length, and weight-for-age were below minus three (− 3.0) Standard Deviations (SD) (i.e., HAZ/LAZ < −3SD; WHZ/WLZ < −3SD; WAZ < −3SD) below the respective median of the World Health Organization (WHO) reference population, respectively [18]. Children without any anthropometric failure from B to G, that is, with moderate undernutrition and/or normal children were categorized as ‘no severe failure’.

### Independent variables

Independent variables were selected based on the previously identified socioeconomic factors [5, 11, 17, 19, 20]. These variables include maternal age in years (15–19, 20–24, 25–29, 30–34, 35–39, ≥40); parents’ education (both parents uneducated, only father was uneducated when mother was educated, only mother was uneducated when father was educated, both parents educated); mother’s current income-earning status (not working, currently working); underweight mother (if the Body Mass Index (BMI) is below 18.5 kgm^− 2^, then ‘yes’; if BMI ≥ 18.5 kgm^− 2^, then ‘no’); mothers received antenatal care (no, yes); received postnatal care (no, yes); mother’s attitudes toward wife-beating (‘justified’ if mothers agree that a husband is justified in hitting or beating his wife if she went out without telling him or/and neglected the children or/and she ever argued with her partner or/and burned food or/and refused to have sex; otherwise ‘not justified’) [8]; decision-making autonomy (‘participated’, if women make decisions alone or jointly with their husband in all three of the following areas: (1) their own health care, (2) major household purchases, and (3) visits to their family or relatives; otherwise, ‘not participated’) [8]; religion (Islam, others); source of drinking water (improved, unimproved) [8], cooking fuel [‘solid fuel’ (including charcoal, wood, straw/shrubs/grass, agricultural crops, and animal dung), and ‘clean fuel’ (including electricity and LPG/natural gas/biogas)] [8]; mass media exposure (no, yes: exposure to either radio, television, newspapers, or magazines at least once a week), wealth index (integrating household asset ownership and access to drinking water and sanitation) [8]. Moreover, factors related to children, such as age, birth order, and birth weight status [21, 22], recent morbidity status (child had at least one morbid condition out of diarrhea, fever and cough in the two weeks preceding the survey) [8] were included. The detailed construction procedures of all independent variables were tabulated in the Additional Table 1.

### Statistical analysis

Socioeconomic characteristics were analysed using descriptive statistics. Bivariate association was used to determine the proportional differences of variables between non-severe undernutrition and severe undernutrition group. The significance level for bivariate analysis was set at *P* < 0.10 (2-tailed). Independent variables that were significant in bivariate analysis were simultaneously entered into the logistic regression models for the adjustment to identify the associated socioeconomic factors of severe undernutrition, measured by CISAF. Results were derived using a stepwise backward selection of regression and expressed by the adjusted odds ratio (AOR) and 95% confidence interval (95% CI). The significance level for logistic regression analysis was set at *P* < 0.05. To control the effect of the complex survey design, all the analyses of this study were performed using Stata’s ‘SVY’ command. Variance Inflation Factor (VIF) was used to evaluate the possible collinearity. However, there was no multicollinearity problem among the variables. All statistical analyses were performed in Stata version 14.2 (StataCorp LP, College Station, Texas).

## Results

### Background characteristics

Of the 7661 under-5 children, about two-thirds lived in rural areas. Three-quarters of the mothers were aged below 30. In rural areas, families were comparatively least wealthy than the urban families (rural 54.3% vs. urban 19.4%), and more mothers were underweight (rural 16% vs. urban 12.1%). In approximately 82% of cases, both parents were educated, whereas nearly 4% of parents were uneducated, regardless of urban-rural settlement. Access to mass media was higher in urban areas (rural 56% vs. urban 79%). Around 40% of the children were aged under 23 months. More than half of the children were not weighted during their birth. And nearly half of the children (47.2%) had recently suffered from any morbidity condition (Table 2).

**Table 2:** Background characteristics of the children

Characteristics	Total (%)^e^	Rural (%)^e^	Urban (%)^e^
**Maternal age (years)**			
15–19	938 (12.2)	657 (13.0)	281 (10.8)
20–24	2679 (35.0)	1818 (36.0)	861 (33.0)
25–29	2146 (28.0)	1395 (27.6)	751 (28.8)
30–34	1293 (16.9)	825 (16.3)	468 (18.0)
34–39	481 (6.3)	291 (5.8)	190 (7.3)
≥ 40	124 (1.6)	70 (1.4)	54 (2.1)
**Parents’ education**			
Both parents were uneducated	294 (3.8)	208 (4.1)	86 (3.3)
Only father was uneducated	865 (11.3)	654 (12.9)	211 (8.1)
Only mother was uneducated	243 (3.2)	154 (3.1)	89 (3.4)
Both parents were educated	6259 (81.7)	4040 (79.9)	2219 (85.2)
**Mother’s income-earning status**			
Not working	4560 (59.5)	2788 (55.1)	1772 (68.0)
Currently working	3101 (40.5)	2268 (44.9)	833 (32.0)
**Underweight mother** (< 18.5 kg/m^2^)			
No	6535 (85.3)	4246 (84.0)	2289 (87.9)
Yes	1126 (14.7)	810 (16.0)	316 (12.1)
**Mothers received antenatal care** (*n* = 4540)			
No	363 (8.0)	285 (9.5)	78 (5.1)
Yes	4177 (92.0)	2727 (90.5)	1450 (94.9)
**Mothers received postnatal care** (*n* = 4535)			
No	1509 (33.3)	970 (32.2)	539 (35.4)
Yes	3026 (66.7)	2041 (67.8)	985 (64.6)
**Mother’s attitudes toward wife-beating**			
Not justified	6274 (81.9)	4042 (79.9)	2232 (85.7)
Justified	1387 (18.1)	1014 (20.1)	373 (14.3)
**Mothers’ decision-making autonomy**^a^			
Not participated	1083 (14.1)	786 (15.5)	297 (11.4)
Participated	6578 (85.9)	4270 (84.5)	2308 (71.9)
**Religion**			
Islam	6997 (91.3)	4608 (91.1)	2389 (91.7)
Others	664 (8.7)	448 (8.9)	216 (8.3)
**Source of water**			
Improved	6658 (86.9)	4332 (85.7)	2326 (89.3)
Unimproved	1003 (13.1)	724 (14.3)	279 (10.7)
**Cooking fuel**			
Clean fuel	2218 (29.0)	899 (17.8)	1319 (50.6)
Solid fuel	5443 (71.0)	4157 (82.2)	1286 (49.4)
**Type of toilet facility**			
Improved	4359 (56.9)	2638 (52.2)	1721 (66.1)
Unimproved	3302 (43.1)	2418 (47.8)	884 (33.9)
**Mass media exposure**			
No	2771 (36.1)	2232 (44.2)	539 (20.7)
Yes	4890 (63.8)	2824 (55.8)	2066 (79.3)
**Wealth index**^b^			
Poorest	1708 (22.3)	1422 (28.1)	286 (11.0)
Poorer	1545 (20.2)	1325 (26.2)	220 (8.4)
Middle	1381 (18.0)	1024 (20.2)	357 (13.7)
Richer	1533 (20.0)	827 (16.4)	706 (27.1)
Richest	1494 (19.5)	458 (9.1)	1036 (39.8)
**Children age**			
0–11 months	1673 (21.8)	1120 (22.2)	553 (21.2)
12–23 months	1583 (20.7)	1058 (20.9)	525 (20.1)
24–35 months	1475 (19.3)	973 (19.2)	502 (19.3)
36–47 months	1417 (18.5)	939 (18.6)	478 (18.4)
48–59 months	1513 (19.7)	966 (19.1)	547 (21.0)
**Sex of child**			
Male	3995 (51.2)	2673 (52.9)	1322 (50.7)
Female	3666 (48.8)	2383 (47.1)	1283 (49.3)
**Birth order**			
First	2902 (37.9)	1848 (36.6)	1054 (40.4)
Second	2507 (32.7)	1617 (32.0)	890 (34.2)
Third	1297 (16.9)	893 (17.7)	404 (15.5)
Fourth and above	955 (12.5)	698 (13.8)	257 (9.9)
**Small birth weight**^c^ **(*n*** **= 4735)**			
No	1815 (38.3)	1036 (32.9)	779 (49.2)
Yes	326 (6.9)	190 (6.0)	136 (8.6)
Not weighted	2594 (54.8)	1927 (61.1)	667 (42.2)
**Recent morbidity status**^d^			
No	4043 (52.8)	2636 (52.1)	1407 (54.0)
Yes	3618 (47.2)	2420 (47.9)	1198 (46.0)
**Total**	7661 (100.0)	5056 (66.0)	2605 (34.0)

^a^defined as women’s decision making power relative to their male partners

^b^integrating household asset ownership and access to drinking water and sanitation

^c^child’s size and weight at birth based on a mother’s perception

^d^child had at least one morbid condition out of diarrhea, fever and cough in the 2 weeks preceding the survey

^e^percentages were weighted

### Prevalence of severe undernutrition among the children under-5

**Fig. 3:**
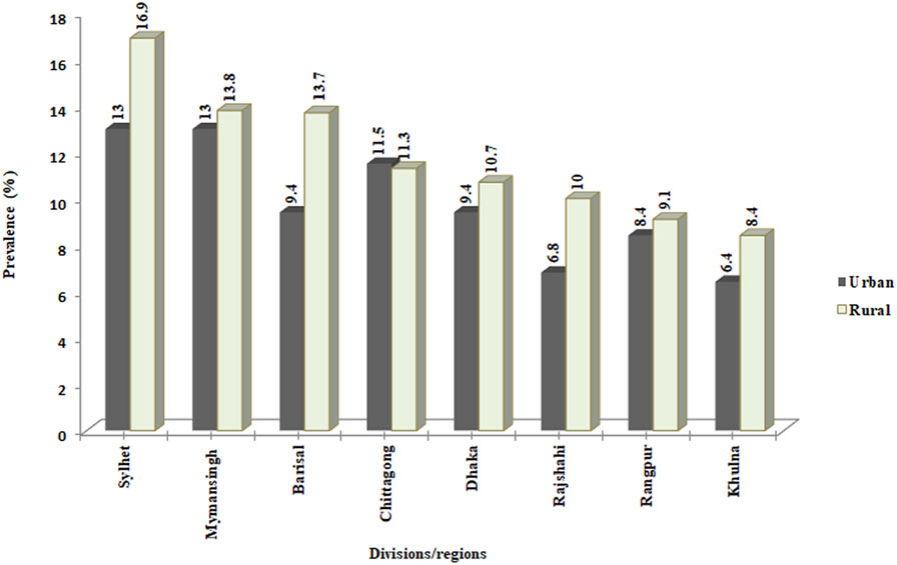
Regional prevalence in urban-rural context

The overall prevalence of severe undernutrition measured by CISAF among the children under-5 was 11.0% in Bangladesh (rural 11.5% vs. urban 9.6%) (Table 3). Figure 2 shows that the overall prevalences of severe stunting, severe wasting, and severe underweight were 9%, 1.5%, and 4.1%, respectively. The prevalence of severe stunting (9.7% vs. 7.7%) and severe underweight (4.2% vs. 3.7%) was higher in rural areas than in the urban (Fig. 2). In terms of regional prevalence, the Sylhet region reported the highest prevalence of severe under-5 child undernutrition in both rural (16.9%) and urban areas (13%) (Fig. 3).

**Table 3:** Proportion of severe under-5 child undernutrition in urban-rural context with respective to different socioeconomic characteristics

Characteristics	Rural		Urban	
	Number	Prevalence (95% CI)	Number	Prevalence (95% CI)
**Maternal age (years)**				
15–19	85	11.5 (9.2, 14.2)	36	13.2 (8.9, 19.1)
20–24	201	10.5 (8.9, 12.3)	77	9.5 (6.9, 12.9)
25–29	176	12.3 (10.4, 14.5)	68	7.9 (6.0, 10.4)
30–34	103	12.4 (9.9, 15.5)	41	8.6 (5.8, 12.6)
34–39	35	11.0 (7.9, 15.1)	84	13.9 (8.8, 21.1)
≥ 40	9	11.8 (6.1, 21.7)	7	9.8 (3.8, 23.1)
	χ^2^ = 3.58, *p* = 0.669		χ^2^ = 11.09, *p* = 0.186	
**Parents’ education**				
Both parents uneducated	42	21.5 (15.7, 28.8)	23	25.9 (17.2, 36.9)
Only father uneducated	113	16.9 (13.7, 20.5)	28	13.9 (9.0, 21.0)
Only mother uneducated	35	21.8 (15.5, 29.6)	11	12.0 (6.1, 22.1)
Both parents educated	419	9.8 (11.3, 14.9)	194	8.4 (6.9, 10.2)
	χ^2^ = 64.76, *p* < 0.001		χ^2^ = 37.89, *p* < 0.001	
**Mother’s income-earning status**				
Not working	321	10.7 (9.2, 12.3)	166	8.8 (7.1, 10.8)
Currently working	288	12.6 (11.1, 14.2)	90	11.4 (8.9, 14.6)
	χ^2^ = 4.40, *p* = 0.080		χ^2^ = 4.52, *p* = 0.060	
**Underweight mother**				
No	468	10.5 (9.4, 11.8)	201	8.9 (7.3, 10.7)
Yes	141	17.1 (14.4, 20.2)	55	15.4 (11.1, 21.0)
	χ^2^ = 26.99, *p* < 0.001		χ^2^ = 12.99, *p* = 0.001	
**Mothers received antenatal care**				
No	49	17.8 (13.1, 23.8)	17	20.9 (11.8, 34.3)
Yes	325	11.2 (9.9, 12.5)	147	9.9 (8.1, 12.0)
	χ^2^ = 10.35, *p* = 0.003		χ^2^ = 10.12, *p* = 0.012	
**Mothers received postnatal care**				
No	92	9.5 (7.6, 11.9)	40	5.9 (4.0, 8.8)
Yes	282	12.9 (11.4, 14.6)	123	13.1 (10.3, 16.5)
	χ^2^ = 7.30, *p* = 0.015		χ^2^ = 19.57, *p* = 0.001	
**Mother’s attitudes toward wife-beating**				
Not justified	458	10.9 (9.7, 12.2)	220	9.7 (8.0, 11.7)
Justified	151	13.9 (11.5, 16.6)	36	9.1 (5.8, 14.0)
	χ^2^ = 7.10, *p* = 0.020		χ^2^ = 0.14, *p* = 0.775	
**Mothers’ decision-making autonomy**^a^				
Not participated	96	11.3 (9.2, 13.8)	36	11.0 (7.8, 15.3)
Participated	513	11.5 (10.3, 12.9)	220	9.4 (7.7, 11.4)
	χ^2^ = 0.03, *p* = 0.865		χ^2^ = 0.78, *p* = 0.390	
**Religion**				
Islam	557	11.5 (10.4, 12.8)	236	9.7 (8.0, 11.7)
Others	52	11.0 (8.5, 14.2)	20	8.5 (4.6, 15.1)
	χ^2^ = 0.12, *p* = 0.726		χ^2^ = 0.28, *p* = 0.676	
**Source of drinking water**				
Improved	532	11.8 (10.6, 13.1)	229	9.6 (8.0, 11.6)
Unimproved	77	9.6 (7.5, 12.1)	27	9.3 (15.0, 20.7)
	χ^2^ = 3.03, *p* = 0.102		χ^2^ = 0.03, *p* = 0.862	
**Cooking fuel**				
Clean fuel	82	8.6 (6.6, 11.1)	95	7.9 (6.2, 10.1)
Solid fuel	527	12.2 (10.9, 13.5)	161	12.1 (9.7, 15.1)
	χ^2^ = 9.98, *p* = 0.011		χ^2^ = 12.99, *p* = 0.005	
**Type of toilet facility**				
Improved	272	10.2 (8.8, 11.7)	146	8.4 (6.8, 10.5)
Unimproved	337	13.0 (11.5, 14.6)	110	12.0 (8.9, 16.0)
	χ^2^ = 9.73, *p* = 0.004		χ^2^ = 8.35, *p* = 0.057	
**Mass media exposure**^b^				
No	328	14.6 (12.7, 16.7)	82	15.4 (10.8, 21.4)
Yes	281	9.3 (8.2, 10.7)	174	8.3 (7.0, 9.8)
	χ^2^ = 33.16, *p* < 0.001		χ^2^ = 23.01, *p* = 0.008	
**Wealth index**^c^				
Poorest	234	16.1 (14.0, 18.4)	50	18.7 (11.7, 28.7)
Poorer	178	12.8 (10.9, 15.1)	40	16.1 (11.6, 22.1)
Middle	98	9.2 (7.3, 11.6)	41	10.6 (7.2, 15.4)
Richer	75	8.9 (6.8, 11.6)	63	9.7 (7.1, 13.1)
Richest	24	5.0 (3.1, 8.1)	62	6.4 (4.9, 8.3)
	χ^2^ = 60.34, *p* < 0.001		χ^2^ = 44.82, *p* = < 0.001	
**Children age**				
0–11 months	95	8.1 (6.6, 10.0)	53	7.5 (5.0, 11.0)
12–23 months	153	13.4 (11.3, 15.8)	56	10.4 (7.6, 14.1)
24–35 months	155	15.2 (12.8, 17.9)	62	14.2 (10.8, 18.6)
36–47 months	102	10.8 (8.6, 13.5)	41	8.2 (5.6, 12.0)
48–59 months	104	10.1 (8.1, 12.6)	44	7.9 (5.5, 11.3)
	χ^2^ = 31.49, *p* < 0.001		χ^2^ = 18.48, *p =* 0.013	
**Sex of child**				
Male	326	11.4 (10.1, 12.9)	137	10.5 (8.5, 12.8)
Female	283	11.6 (10.0, 13.4)	119	8.7 (6.8, 11.1)
	χ^2^ = 0.03, *p* = 0.871		χ^2^ = 2.29, *p =* 0.159	
**Birth order**				
First	197	10.1 (8.6, 11.8)	84	8.3 (6.4, 10.9)
Second	184	10.7 (9.1, 12.4)	83	8.7 (6.5, 11.7)
Third	114	12.3 (10.1, 14.8)	41	9.3 (6.5, 13.0)
Fourth and above	114	16.2 (12.8, 20.3)	48	18.9 (14.1, 24.8)
	χ^2^ = 19.99, *p* = 0.001		χ^2^ = 27.42, *p* = 0.002	
**Small birth weight**^d^				
No	74	6.6 (5.2, 8.4)	47	6.6 (4.8, 9.0)
Yes	32	18.3 (12.8, 25.3)	29	18.3 (11.8, 27.1)
	χ^2^ = 48.54, *p* < 0.001		χ^2^ = 29.06, *p* = 0.003	
**Recent morbidity status**^e^				
No	112	11.2 (9.8, 12.7)	147	10.4 (8.4, 12.9)
Yes	297	11.8 (10.4, 13.5)	109	8.7 (6.7, 11.1)
	χ^2^ = 0.53, *p* = 0.495		χ^2^ = 2.32, *p* = 0.206	
**Total**	609	11.5 (10.4, 12.7)	256	9.6 (8.0, 11.5)

^a^defined as women’s decision-making power relative to their male partners

^b^exposure to either radio, television, newspapers, or magazines at least once a week

^c^integrating household asset ownership and access to drinking water and sanitation

^d^child’s size and weight at birth based on a mother’s perception

^e^child had at least one morbid condition out of diarrhea, fever and cough in the 2 weeks preceding the survey

*p* values are obtained from Chi-square estimation

From the bivariate association, in both urban and rural areas, children of uneducated parents (urban: 25.9% vs 8.4%; rural: 21.5% vs 9.8%), underweight mothers (urban: 15.4% vs 8.9%; rural: 17.1% vs 10.5%), poorest households (urban: 18.7% vs 6.4%; rural: 16.1% vs 5.0%), and with small birth weight (urban: 18.3% vs 6.6%; rural:) reported a higher prevalence of severe under-5 undernutrition than the children of educated parents, normal BMI mothers, richest households, and normal birth weight (Table 3).

### Socioeconomic factors associated with severe under-5 undernutrition

The key associated factors for under-5 undernutrition in urban areas were: children born with small birth weight (Adjusted Odds Ratio, AOR: 3.99, 95% CI: 2.12, 7.52) vs. healthy weight; children of parents with no formal education (AOR: 2.34, 95% CI: 1.27, 4.32) vs. children of educated parents; mothers did not receive postnatal care (AOR: 2.13, 95% CI: 1.32, 3.44) vs. the recipient mothers; children’s birth order ≥4 (AOR: 1.75, 95% CI: 1.97, 2.86) vs. children’s first birth order; poorest children (AOR: 2.40, 95% CI: 1.47, 3.93) vs. the richest; and children of underweight mothers (AOR: 1.58, 95% CI: 1.04, 2.40) vs. normal mothers (Table 4).

**Table 4:** Association between socioeconomic characteristics and under-5 severe child undernutrition in rural-urban context

Socioeconomic characteristics	Rural		Urban	
	Adjusted Odds Ratio (95% CI)	P values	Adjusted Odds Ratio (95% CI)	P values
**Parents’ education** ^**a**^				
Both parents uneducated	1.92 (1.35–2.73)	< 0.001	2.34 (1.27–4.32)	0.006
Only father uneducated	1.54 (1.23–1.94)	< 0.001	1.42 (0.86–2.36)	0.170
Only mother uneducated	2.15 (1.46–3.18)	< 0.001	1.08 (0.51–2.29)	0.843
Both parents educated (RC)	1.00		1.00	
**Underweight mother** (< 18.5 kg/m2) ^a^				
No (RC)	1.00		1.00	
Yes	1.50 (1.21–1.84)	< 0.001	1.58 (1.04–2.40)	0.033
**Mothers received antenatal care** ^**b**^				
No	1.28 (1.00–1.64)	0.050		
Yes (RC)	1.00			
**Mothers received postnatal care** ^**b**^				
No			2.13 (1.32–3.44)	0.002
Yes (RC)			1.00	
**Mother’s attitudes toward wife-beating** ^**a**^				
Not justified	1.00			
Justified	1.25 (1.02–1.52)	0.028		
**Mass media exposure** ^**a,c**^				
No	1.25 (1.04–1.51)	0.018		
Yes (RC)	1.00			
**Wealth index** ^**a,d**^				
Poorest	2.44 (1.57–3.78)	< 0.001	2.40 (1.47–3.93)	< 0.001
Poorer	2.20 (1.43–3.38)	< 0.001	2.35 (1.34–4.11)	0.003
Middle	1.68 (1.09–2.61)	0.020	1.49 (0.90–2.46)	0.122
Richer	1.80 (1.15–2.82)	0.011	1.36 (0.91–2.02)	0.132
Richest (RC)	1.00		1.00	
**Children age** ^**a**^				
0–11 months (RC)	1.00		1.00	
12–23 months	1.78 (1.36–2.33)	< 0.001	1.46 (0.89–2.39)	0.131
24–35 months	2.10 (1.61–2.74)	< 0.001	2.19 (1.37–3.51)	0.001
36–47 months	1.37 (1.03–1.83)	0.030	1.16 (0.69–1.96)	0.577
48–59 months	1.21 (0.90–1.61)	0.202	1.08 (0.64–1.83)	0.765
**Birth order** ^**a**^				
First (RC)			1.00	
Second			1.07 (0.74–1.54)	0.728
Third			1.05 (0.65–1.69)	0.833
Fourth and above			1.75 (1.07–2.86)	0.027
**Small birth weight** ^**b,e**^				
No (RC)	1.00		1.00	
Yes	2.84 (1.88–4.30)	< 0.001	3.99 (2.12–7.52)	< 0.001

RC, reference category

^**a**^ model adjusted with all variables found significant in the bivariate analysis except mothers received antenatal care, mothers received postnatal care, and small birth weight

^**b**^ model adjusted with all variables found significant in the bivariate analysis including mothers received antenatal care, mothers received postnatal care and small birth weight

^**c**^ exposure to either radio, television, newspapers, or magazines at least once a week

^**d**^ integrating household asset ownership and access to drinking water and sanitation

^**e**^ child’s weight at birth based on measuring and mother’s perception

Results are derived using stepwise backward selection of regression

On the other hand, in the rural areas, children born with small birth weight (AOR: 2.84, 95% CI: 1.88, 4.30) vs. healthy weight; children who lived in poorest households (AOR: 2.44, 95% CI: 1.57, 3.78) vs. the richest households; children aged less than 36 months (for age group 24–35 months (AOR: 2.10, 95% CI: 1.61, 2.74); for age group 12–23 months (AOR: 1.78, 95% CI: 1.36, 2.33)) vs. children of age group 0–11 months; children of mothers with no formal education (when fathers are educated) (AOR: 2.15, 95% CI: 1.46, 3.18) vs. children of educated parents, etc. socioeconomic factors showed a positive association with severe under-5 undernutrition (Table 4).

## Discussion

To the best of our knowledge, this is the first study in Bangladesh that used the updated version of the CIAF (i.e., the CISAF) to estimate the prevalence of severe under-5 undernutrition and its associated socioeconomic factors. Though the three most conventionally used stunting, wasting, and underweight indicators represent different physiological manifestations of undernutrition, they all share similar causal factors [23]. Individual assessments of stunting, wasting, and underweight underestimate the total burden of undernutrition. Since children may suffer from more than one type of undernutrition, only the data on prevalence may not provide a convincing estimate of the proportion of undernourished children in the population. Here the application of CIASF identifies all malnourished children and provides a single estimate of the burden of severe undernutrition among the children under age 5 [15].

In this study, we used the aggregated CISAF measurement to investigate severe under-5 child undernutrition in Bangladesh. More than one out of 10 children under-5 (11%) has been found to suffer from severe undernutrition. In a press release of UNICEF (2020), the prevalence of moderate to severe underweight and stunting was found to be about 23 and 28% in 2019, respectively [24]. Similarly, other organizations in Bangladesh (such as icddr,b, and Save the Children) working on child undernutrition have recently reported a similar prevalence rate of either severe stunting or underweight. But none of them used such a composite method to measure severe underweight, i.e., CISAF. So, the actual composite scenario of severe undernutrition among the under-5 children in Bangladesh has been overlooked. Severe stunting, underweight or child undernutrition in Bangladesh was investigated in different studies. Our research results are in line with these previous studies that rural under-5 children experience severe undernutrition than the urban ones [25–28]. But the composite index for measuring severe undernutrition, that is CISAF was not applied in those studies.

On the contrary, according to a few studies, severe under-5 undernutrition was more prevalent in the urban areas of Bangladesh [29, 30]. It is noteworthy that these studies [29, 30] used the data of the last two decades. There has been a significant change in the urban areas of Bangladesh for the last few years, such as universal access to primary education, improving the household socioeconomic status, adequate maternal healthcare utilization, improvement of transportation facilities, and awareness about nutrition, which ultimately improved the overall nutritional status in the urban areas [27–29]. Compared to this, rural areas did not get the touch of such extensive development in Bangladesh. It might be one of the plausible explanations behind such a higher prevalence of severe under-5 undernutrition among the rural children of Bangladesh.

The prevalence of severe under-5 child undernutrition was more dominant among the children of uneducated parents. One in four children of the uneducated parents experienced severe undernutrition regardless of being born in rural or urban areas. Further, low birth weight children had a greater odd of being severely malnourished irrespective of the rural or urban context. For example, in our study, children with low birth weight were more likely to experience severe undernutrition (3.99 times the odds) living in the urban areas than rural children with low birth weight (2.84 times the odds). Also, one-fifth of the under-5 children born in rural and urban areas with a small birth weight experienced severe undernutrition. These findings were matched with the previous studies of Pakistan, Nepal, Malawi, Mexico, and Iran, which reported that children born with small birth weight were more likely to experience different types of undernutrition [19, 31–34]. Generally, children born with a low birth weight gain inadequate amounts of height and weight [35]. Thus, they may remain shorter and lighter, and might suffer from severe undernutrition without adequate nutritional support. Additionally, children with small birth weights are often born to households with low socioeconomic status and mothers with poor health conditions [36]. Due to the irregular distribution of food for children in poverty-stricken households, and the knowledge gap of parents/caregivers for adequate micronutrient supplementation, children can be deprived of nutritious food intake and eventually suffer from acute undernutrition [12, 14]. Again, parental illiteracy is frequently connected with low birth weight and other variables, such as inadequate maternal healthcare access and child caregiving. All these interlinked factors are substantially responsible for the poor nutritional outcomes of mothers and the low birth weight among the children [37, 38].

Poor household status has been found as one of the strongly associated factors for severe undernutrition among under-5 children in several studies [5, 39, 40], which is matched with our findings. Nearly one-fifth of the children who lived in the least wealthy households experienced severe under-5 undernutrition, regardless of the urban-rural context of this study. The odds of severe under-5 undernutrition were 2.44 times higher for children living in rural areas and 2.40 folds higher for urban children from the poorest wealth index. Poor parents often cannot afford a minimum diet and proper postnatal care for their children [41]. Other plausible explanations, such as increased clustering of urban poor in slums with limited access to public health and nutrition services and amenities, high population density, poor quality drinking water, inadequate sanitation facilities, are also blameworthy for the higher prevalence of severeundernutrition among the under-5 children of the poor households [42].

In this study, uneducated parents were one of the strongly associated factors of severe child undernutrition, especially in rural areas. Educated parents were found to be significantly associated with better nutritional conditions during pregnancy and after birth and had been an indirect predictor of better child health throughout life [43, 44]. According to the studies from Bangladesh [43] and Pakistan [44], the likelihood of child undernutrition increased when their parents were uneducated, which is consistent with our study findings in rural areas. Again, maternal education could be an important determinant of child survival in rural areas since the child mortality and morbidity rates are usually high [45]. Like our study finding, maternal lower educational status in rural areas increases the odds of severe child undernutrition. Hence, a greater focus on parental education facilities is required irrespective of geographical regions to accelerate the nationwide improvements in child nutrition status.

Children’s birth order ≥4 were 1.75 times more likely to experience severely malnourished living in urban areas. Studies from Bangladesh and India showed that children in higher birth order were more likely to be severely malnourished in Bangladesh’s urban areas [25] but in rural areas of India [46]. Previous studies from Bangladesh [5], Congo [47], and Ethiopia [48] also reported that children with higher birth order were more likely to experience undernutrition regardless of urban-rural context. When the number of children increases, household members get competitive for foods, and the equality of providing necessary foods could not be maintained for all children [49]. The risk of severe undernutrition is usually high in older children (i.e., age 4 to 5 years) in Nepal, Pakistan, Ethiopia, and Congo [17, 48, 50, 51].

In comparison, severe undernutrition is high in younger (age 1 to 2 years) children in India [52], indicating this problem’s complex nature. We found that toddlers (age 2 to 3 years) living in rural areas had higher odds of severe under-5 undernutrition than toddlers living in urban areas. A similar level of provision of health and nutritional care available for urban children might be the reason for the insignificant association between children’s age and severe undernutrition. At 6–36 months, inappropriate feeding behaviors and other factors (e.g., infection and food shortage) may be responsible for one-third of undernutrition cases, depending on population, place, time, and season [53]. In addition, lack of attention in rural areas (urban-rural disparities) in receiving complementary feeding, access to health services, preventive and curative interventions influence nutrition outcomes [54].

Severeundernutrition is a multifaceted, complex phenomenon, involving many immediate causes (e.g., poor diet habits, diarrhea, ages of breastfeeding children) and underlying causes (e.g., income inequality, food insecurity, access to safe water, environmental hygiene) [55, 56]. The risk difference between most affluent and poorest was higher among children in rural areas than in urban areas, indicating a greater rich-poor gap. Such socioeconomic inequality can be reduced by increasing income-generating activities driven by public and private entities and introducing different microfinance programs. These endeavors need to be aimed at deprived and vulnerable individuals and ensure their participation with a standard wage structure under the national nutritional security system. In Ethiopia, improved per capita household income increased available funds for food expenditure and basic health care needs and developed children’s nutritional status [57]. Moreover, universal education and standard health care should be available and accessible to all women, especially in rural and remote areas. Improving access to community-based education and standard health care to mother include conferring many benefits from improved caregiver practices; enhancing health and environmental knowledge; increasing educated and skilled workforce; living in better neighborhoods, reducing gender-based violence, child marriage, and early childbearing; and reducing maternal death rates in terms of improved maternal and child nutritional status [58].

### Limitations

The BDHS 2017–18 data used for this study was one of Bangladesh’s largest nationally representative samples. The stability of the data set allows changes over time to be monitored with some confidence. However, there were some limitations too for this study. First, we could not use the infant and young children feeding data to the models because the data available in the BDHS were only for the children aged 6 to 23 months. But our study considered children under age 5. So, there would be too many missing variables, and this study would be limited only to children under age 2. Further, data on potential confounders like diet, food insecurity, and parents smoking behavior were unavailable. Secondly, the cross-sectional data was insufficient to establish a causal relationship, consequently limiting the findings’ applicability. Thirdly, the BDHS data were collected retrospectively and self-reported, thus subject to underreporting, information bias, and recall bias. However, data were collected using validated tools and standard procedures. Using seven nutritional status measurements, CISAF provided a credible estimate of the overall proportion of severe under-5 child undernutrition and the complex interplay between individual, community, public policy, and environment level associated factors.

## Conclusion

The overall prevalence of severe under-5 child undernutrition was greater than conventional indicators. One in ten children living in both rural and urban areas experience severe undernutrition. Rural under-5 children were experiencing more severe undernutrition than the urban ones. Children of uneducated parents, underweight mothers, living in the poorest households, and with small birth weight experienced severe undernutrition regardless of urban-rural setting. Rural areas were identified as a greater rich-poor gap. The findings of this study imply that more research should be conducted from different aspects using such composite index (like- CISAF) to depict the comprehensive scenario of severe undernutrition among the under-5 children in Bangladesh. From a policy-making perspective, only the educational attainment and access to health and nutritional care may not be enough to reduce the burden of severe under-5 child undernutrition in rural settings due to the complex interplay between the socioeconomic and cultural factors. Bangladesh needs to focus on household wealth generation by introducing intervening microfinance programs, improving maternal health through proper maternal healthcare utilization, and different intervention programs on vulnerable groups, including the children from the poorest socioeconomic strata or children in the urban areas. Additionally, strengthening the role of parents through awareness programs and yard meetings to improve their knowledge in different aspects like- family planning, reduction of fertility, child nutrition, and maternal care. Furthermore, to achieve the SDGs by 2030, a targeted multi-sectoral healthcare program is required to reduce the prevalence of short-statured children at an early age, to advance knowledge on complementary foods for young children, and to reinforce family planning programs aimed at increasing the age at birth and decreasing higher-order births.

## Supplementary Information

**Additional file 1.**

## Data Availability

The data underlying the results presented in the study are publicly accessible and available from the DHS website (https://dhsprogram.com/data/available-datasets.cfm). The name of the dataset is Bangladesh Demographic and Health Survey (BDHS).

## Abbreviations

AOR: Adjusted Odds Ratio
BDHS: Bangladesh Demographic Health Survey
CI: Confidence Interval
CIAF: Composite Index of Anthropometric Failure
CISAF: Composite Index of Severe Anthropometric Failure
EA: Enumeration Area
LPG: Liquid Petroleum Gas
PSU: Primary Sampling Unit
SDG: Sustainable Development Goal
WHO: World Health Organization
