# Supplementary material for: Urban-rural differences in the associated factors of severe under-5 child undernutrition based on the composite index of severe anthropometric failure (CISAF) in Bangladesh
Source: BMC Public Health. 2021 Nov 23;21:2147. doi: 10.1186/s12889-021-12038-3 (PMC8611976; doi:10.1186/s12889-021-12038-3)
Supplement: Supplementary file 1 — Additional file 1. [file 12889_2021_12038_MOESM1_ESM.docx]

**Additional file 1**

**Table S1.** Measurements of independent variables

| **Variable** | **Description** | **Measurement** | **Scale of measurement** |
| --- | --- | --- | --- |
| **Mothers’ age (in years)** | Age of mothers at the time of data collection | 15-19, 20-24, 25-29, 30-34, 35-39, ≥ 40 | Discrete, categorial |
| **Parents’ education** | Parental educational status. Accomplishment of at least five years of schooling (primary level, class 1 to 5) refers educated. No (0 years of schooling) schooling year refers uneducated. | Both parents were uneducated, only father was uneducated, only mother was uneducated, both parents were educated | Categorical |
| **Mother’s income-earning status** | Mother engaged in economic activity at the time of data collection | Not working,  Currently working | Binary |
| **Underweight mother** | Mother with <18.5 kg/m^2^ of body mass index refers to underweight, and ≥18.5 kg/m^2^ refers to healthy or not underweight | No, Yes | Binary |
| **Mothers received antenatal care** | At least one medical surveillance and review performed during pregnancy for the early detection of possible complications of pregnancy | No, Yes | Binary |
| **Mothers received postnatal care** | At least one postnatal care is the individualized care provided to meet the needs of a mother and her baby following childbirth | No, Yes | Binary |
| **Mother’s attitudes toward wife-beating** | Respondents are asked if they agree that a husband is justified in hitting or beating his wife under each of the following five circumstances: she burns the  food, she argues with him, she goes out without telling him, she neglects the children, and she refuses to have sex with him. If respondents answer “yes” in  at least one circumstance, they are considered to have attitudes justifying wife beating. | Justified, Not justified | Binary |
| **Mothers’ decision-making autonomy** | In the BDHS surveys, a woman’s decision-making power is assessed on the following three themes: 1) a woman who usually decides on her healthcare 2) a woman who usually decides on large household purchases and 3) a woman who usually decides on visits to family or relatives. The response options were as follows: (a) respondent alone, (b) respondent and husband/partner, (c) respondent and another person, (d) husband/partner alone, (e) someone else, (f) other. For each question, a value of 1 was assigned for inability in decision-making if the responses were d, e, or f and 0 for otherwise if the responses were a, b, or c. The values were then added, resulting in a score from 0 to 3. The Cronbach’s α for the instruments was 0·79, indicating high internal consistency. | Not participated, Participated | Binary |
| **Religion** | Religious belief of mothers. Such as, Islam, Hindu, Christian and Buddhist | Islam, others | Binary |
| **Source of water** | Improved: piped into dwelling, piped to yard/plot, public tap/standpipe, piped to neighbor, tube well or borehole, protected well, protected spring, rainwater, tanker truck, cart with small tank, bottled water; unimproved: unprotected well, unprotected spring, surface water (river, dam, lake, pond, stream, canal, irrigation channel), other | Improved, unimproved | Binary |
| **Type of toilet facility** | Improved: flush - to piped sewer system, flush - to septic tank, flush - to pit latrine, flush - don't know where, pit latrine - ventilated improved pit, pit latrine - with slab, composting toilet; unimproved: flush - to somewhere else, pit latrine - without slab / open pit, bucket toilet, hanging toilet/latrine, other | Improved, unimproved | Binary |
| **Cooking fuel** | Solid fuel includes coal, lignite, charcoal, wood, straw / shrubs / grass, agricultural crop and animal dung; Clean fuel includes electricity, natural gas, processed gas, biogas, kerosene | Clean fuel, Solid fuel | Binary |
| **Mass media exposure** | Mass media exposure through television, radio and newspaper/magazine has been defined as exposure to at least one media that exposes to at least once a week | No, Yes | Binary |
| **Wealth index** | Wealth index in the DHS surveys is calculated, by the DHS authority, based on information on household characteristics and assets using principal component analysis. Then households are classified into quintiles based on the values of the wealth index, where households with lower values of the index is considered as poorest and vice-versa | Poorest, poorer, middle, richer, richest | Categorical |
| **Children’s age (in months)** | Age of the children at the time of data collection | 0-11 months, 12-23 months, 24-35 months, 36-47 months, 48-59 months | Categorical |
| **Sex of child** | Sex differential of children | Male, Female | Binary |
| **Birth order** | Birth order is the chronological order of sibling births in a family | One, two, three, four and above | Categorical |
| **Low birth weight** | Children were <2.5 kg of weight during birth. Approximately 75% mothers can correctly report their baby's size at birth, therefore mother’s recall is a valid proxy measure of birth weight. | No, Yes, Not weighted | Categorical |
| **Recent morbidity status** | Children had at least cough, fever or diarrhea before 2 weeks of the survey | No, Yes | Binary |
